# Cannabidiol-Induced Autophagy Ameliorates Tau Protein Clearance

**DOI:** 10.1007/s12640-025-00729-3

**Published:** 2025-02-04

**Authors:** Talita A. M. Vrechi, Gabriel C. Guarache, Rafaela Brito Oliveira, Erika da Cruz Guedes, Adolfo G. Erustes, Anderson H. F. F. Leão, Vanessa C. Abílio, Antonio W. Zuardi, Jaime Eduardo C. Hallak, José Alexandre Crippa, Claudia Bincoletto, Rodrigo P. Ureshino, Soraya S. Smaili, Gustavo J. S. Pereira

**Affiliations:** 1 Department of Pharmacology, Escola Paulista de Medicina, Universidade Federal de São Paulo, Rua Três de Maio, 100, São Paulo, SP CEP: 04044-020 Brazil; 2 Department of Cell and Developmental Biology, University College London, London, UK; 3 Department of Biological Sciences, Universidade Federal de São Paulo, Diadema Campus, Diadema, SP Brazil; 4 Laboratory of Molecular and Translational Endocrinology, Escola Paulista de Medicina, Universidade Federal de São Paulo, São Paulo, SP Brazil; 5 National Institute for Translational Medicine (INCT-TM, CNPq), Ribeirão Preto, Brazil; 6 Department of Neuroscience and Behavior, Universidade de São Paulo, USP, Ribeirão Preto, Brazil

**Keywords:** CBD, Tau protein, Autophagy, Neuroprotection

## Abstract

Tau is a neuronal protein that confers stability to microtubules; however, its hyperphosphorylation and accumulation can lead to an impairment of protein degradation pathways, such as autophagy. Autophagy is a lysosomal catabolic process responsible for degrading cytosolic components, being essential for cellular homeostasis and survival. In this context, autophagy modulation has been postulated as a possible therapeutic target for the treatment of neurodegenerative diseases. Studies point to the modulatory and neuroprotective role of the cannabinoid system in neurodegenerative models and here it was investigated the effects of cannabidiol (CBD) on autophagy in a human neuroblastoma strain (SH-SY5Y) that overexpresses the EGFP-Tau WT (Wild Type) protein in an inducible Tet-On system way. The results demonstrated that CBD (100 nM and 10 µM) decreased the expression of AT8 and total tau proteins, activating autophagy, evidenced by increased expression of light chain 3-II (LC3-II) protein and formation of autophagosomes. Furthermore, the cannabinoid compounds CBD, ACEA (CB1 agonist) and GW-405,833 (CB2 agonist) decreased the fluorescence intensity of EGFP-Tau WT; and when chloroquine, an autophagic blocker, was used, there was a reversal in the fluorescence intensity of EGFP-Tau WT with CBD (1 and 10 µM) and GW-405,833 (2 µM), demonstrating the possible participation of autophagy in these groups. Thus, it was possible to conclude that CBD induced autophagy in EGFP-Tau WT cells which increased tau degradation, showing its possible neuroprotective role. Hence, this study may contribute to a better understanding of how cannabinoids can modulate autophagy and present a potential therapeutic target in a neurodegeneration model.

**Graphical Abstract:**

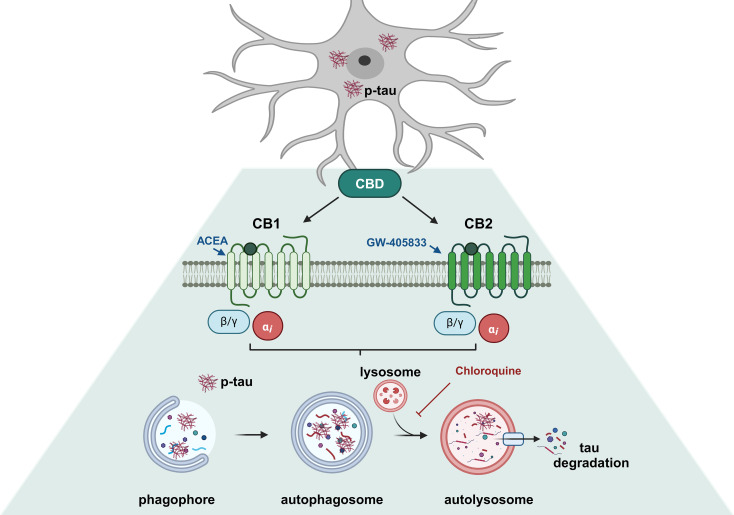

**Supplementary Information:**

The online version contains supplementary material available at 10.1007/s12640-025-00729-3.

## Introduction

Tau is a phosphoprotein predominantly expressed in cells of the central nervous system (CNS) that confers stability to microtubules (Wang and Liu 2008). The excessive phosphorylation, misfolding and aggregation of tau protein cause cellular stress, leading to activation of cell death pathways, and consequently neurodegeneration (Fontaine et al. 2015). Some neurodegenerative diseases can be classified as tauopathies, including those characterized by dementia, such as Alzheimer’s disease (AD), frontotemporal dementia (FTD), Pick’s disease and progressive supranuclear palsy (Goedert and Spillantini 2011). AD is the most common neurodegenerative disease associated with aging and one of the main causes of dementia in the elderly (Castellani et al. 2010). The pathophysiology of AD is characterized by the presence of two distinct biomarkers: (1) amyloid plaques, composed by β-amyloid peptide (βA) that aggregates in extracellular space and (2) neurofibrillary tangles, composed by hyperphosphorylated tau, which leads to cellular stress and cognitive impairment (Fulga et al. 2007). Hyperphosphorylated tau detaches from microtubules disrupting its structure and leading to intracellular accumulation of isolated tau in neurons (Stoothoff and Johnson 2005). In addition, studies suggest that in tauopathies, including AD, there is an impairment of protein degradation pathways, such as autophagy (Nassif and Hetz 2012; Zhang et al. 2017; Festa et al. 2021).

Autophagy is a lysosomal catabolic degradation process based on double-membrane vesicles formation called autophagosomes, and it is essential for survival, development, and maintenance of cellular homeostasis (Yang and Klionsky 2010). The blockage of autophagy can induce tau accumulation and aggregation in neurodegenerative processes, which was observed in in vivo and in vitro models of tauopathies (Liu et al. 2009; Salama et al. 2018; Silva et al. 2019) and in *post-mortem* brains from AD patients (Piras et al. 2016; Long et al. 2020). Studies have demonstrated that autophagy is one of the main pathways involved in tau degradation and that dysfunctions in the autophagy-lysosome system lead to the formation of insoluble tau oligomers and aggregates, while the induction of autophagy can reduce the amount of these aggregates (Congdon et al. 2012; Krüger et al. 2012; Caballero et al. 2021; Hamano et al. 2021). Several studies have shown that the endocannabinoid system can modulate autophagy (Gugliandolo et al. 2020). This system, which includes cannabinoid receptors type 1 (CB1R) and type 2 (CB2R), endogenous cannabinoids, and the enzymes involved in their synthesis and degradation, play a crucial role in maintaining the balance between protein aggregation and clearance. Growing evidence underscores its capacity to regulate autophagic processes, leading to insights into the mechanisms underlying neurodegenerative diseases (Hiebel et al. 2014; Basavarajappa et al. 2017; Gugliandolo et al. 2020).

The deletion for CB1R in mice promoted the pathological accumulation of proteins, which are not degraded by lysosomal enzymes through autophagic flux, influencing the onset and the course of brain aging (Piyanova et al. 2013). The deletion of CB2R impairs the accumulation of the autophagic protein LC3-II in the spinal cord of an experimental model of autoimmune encephalomyelitis in mice, while the activation of CB2R reverted this effect (Shao et al. 2014). Recently, we demonstrated that cannabidiol (CBD) induced autophagy in neural cells through the activation of CB1, CB2 and TRPV1 receptors by regulating the phosphorylation of ERK1/2 and AKT kinases in a mTORC1 independent manner (Vrechi et al. 2021). Thus, the modulation of autophagy by cannabinoids could be considered as a new strategy and a possible approach to deal with neurodegenerative processes, since it also suppresses the accumulation of proteins, eliminating aggregation-prone proteins and damaged organelles that may be involved in neurodegeneration (Hara et al. 2006; Komatsu et al. 2006).

Here we investigated the possible neuroprotective role of CBD in a neuroblastoma cellular model of tauopathy, in an inducible Tet-On system that conditionally overexpresses tau. Our study provides evidence that CBD induces autophagy and reduces the accumulation of tau, which could represent a potential new therapeutic strategy for neurodegeneration.

## Methods

### Drugs

Cannabidiol (CBD) was purchased from BSPG-Pharm (Sandwich, Kent, UK); Arachidonyl-2´-chloroethylamide hydrate (ACEA), 1-(2,3-Dichlorobenzoyl)-5-methoxy-2-methyl-(3-(morpholin-4-yl)ethyl)-1 H-indole hydrochloride (GW-405833), wortmannin, chloroquine, doxycycline hydrochloride hemiethanolate hemihydrate, ammonium chloride and Earle’s Balanced Salt Solution (EBSS) were purchased from Sigma-Aldrich Chemical Co. (St Louis, MO, USA).

### Cell Culture

Human neuroblastoma (SH-SY5Y) cell line was maintained in high glucose Dulbeccos’s modified Eagle’s medium, supplemented with 10% fetal bovine serum and 1% penicillin/streptomycin (Thermo Fisher Scientific) and kept in 37 °C in a humidified CO_2_ atmosphere. The undifferentiated cells used as tauopathy model was previously established and described (Costa et al. 2022). Briefly, cells were transduced using inducible Tet-On conditional expression system, containing the tetracycline-controlled reverse transactivation. Cells were infected with the pLVX-Tight-Puro-EGFP-Tau WT vector, to conditionally express tau. The Tet-On system and the expression of EGFP-Tau(WT) is activated by doxycycline (1 µg/mL) treatment for 72 h.

### Cell Viability Assay

To investigate the neuroprotective role of cannabinoid agonists in a tauopathy cellular model, the SH-SY5Y cell line was transduced with the lentivirus containing the vector pVLX-GFP-Tau0N4R Wild type (WT), as previously described (Costa et al. 2022). Cells were plated and the Tet-On system was activated with doxycycline (1 µg/mL) for 72 h. After this period, cells were treated with CBD (100, 250 nM, 1 and 10 µM), ACEA and GW405833 (100, 250 nM, 1 and 2 µM) for 24 h. Cell viability was performed using the 3-(4,5-Dimethylthiazol-2-yl)-2,5-diphenyltetrazolium bromide (MTT, Sigma-Aldrich) reduction assay. To evaluate cell viability, the culture medium was removed and MTT solution (0.5 mg/mL) in serum-free DMEM was added and incubated at 37 °C for 4 h. The medium was then gently removed, and the formazan crystals were solubilized in dimethyl sulfoxide (DMSO, Sigma-Aldrich). Colorimetric determination of MTT reduction was measured at 570 nm with a reference wavelength at 630 nm in a microplate reader (BioTek). Data are presented as relative expression to control group, normalized as 100%.

### Western Blotting

To assess whether treatment with CBD could reduce the cytosolic levels of tau, the system was activated with doxycycline (1 µg/mL) for 72 h, followed by treatment with CBD (100, 250 nM, 1 and 10 µM) for 24 h. After, cells expressing EGFP-Tau WT and treated with CBD were collected in lysis buffer (150 mM NaCl, 1% NP-40, 0.5% deoxycholic acid, 0.1% SDS, 50 mM Tris pH 8.0, and 2 mM MgCl_2_) supplemented with protease and phosphatase inhibitors cocktails. The insoluble fraction was removed by centrifugation at 15,000 ×g, 4 °C for 10 min and the protein concentration in supernatants was determined using the Bradford assay (Bio-Rad). Samples were prepared in NuPAGE™ LDS Sample Buffer (4X) sample buffer (Thermo Fischer Scientific). Total protein lysates (20–30 µg) were loaded and subjected to SDS-PAGE, transferred to PVDF membranes (Millipore) using a Trans-Blot cell system (Bio-Rad) and blocked for 1 h at room temperature with 5% non-fat dry milk in Tris buffered saline solution containing 1% Tween-20 (TBS-T). Membranes were incubated with primary antibodies overnight at 4 °C: anti-LC3B (Cell Signaling Technology Inc., #2775S, 1:2000,), anti-p-AT8-tau (pSer202/pTrh205, Thermo Fisher Scientific, #MN1020, 1:1000), anti-p-AT180-tau (Trh231, Thermo Fisher Scientific, #MN1040, 1:1000), anti-tau-5 (Abcam, #ab80579, 1:1000) and anti-p62 (MBL #PM045, 1:2000), α-tubulin (Sigma-Aldrich, #T8203, 1:5000) or GAPDH (Sigma-Aldrich, #G8795, 1:5000) as housekeep control. The membranes were incubated with appropriate horseradish peroxidase-conjugate secondary antibody (1:5,000) for 1 h at room temperature. Protein signals were visualized using the Western Lightning Plus-ECL chemiluminescence system (Perkin Elmer) and the luminescence was captured using an Uvitec chemidoc-imaging platform (UVITEC Alliance 4.7, Cambridge). The quantification was performed using the Uvitec Alliance software and the protein bands were normalized relative to the internal control expression. Data are presented as relative expression to control group.

### mCherry-LC3 Puncta Formation

The EGFP-Tau WT cells were plated on 25 mm glass coverlips and treated with doxycycline for 72 h. Cells were transfected as previously described in Costa et al. (2022), using mCherry-LC3 (5 µg) and lipofectamine 3000 reagent (10 µL) (Thermo Fisher Scientific) according to manufacturer’s protocol. After 24 h of plasmid expression, cells were treated with CBD (100 nM and 10 µM) or Earle’s Balanced Salt Solution (EBSS) medium for 2 h. Cells were fixed with 4% paraformaldehyde and coverslips were prepared in microscope slides with Fluoromount (Sigma Aldrich). Images were randomly acquired in a Zeiss LSM 780 confocal microscope (Zeiss LSM 780 Axiovert 200 M, Carl Zeiss) at 63× magnification, using 543/615 nm emission filters, and analyzed using the ImageJ software (NIH).

### Measurement of Tau Clearance

Cells were cultured (1 × 10^4^) in black 96-well plates, followed by the activation of Tet-On system with doxycycline (1 µg/mL) for 72 h. After this period, cells were pretreated with wortmannin (250 nM) and chloroquine (25 µM) for 30 min, followed by the treatments with CBD (100, 250 nM, 1 and 10 µM), ACEA and GW-405,833 (100, 250 nM, 1 and 2 µM) for 24 h. Nuclei were stained with Hoechst 33,342 (2 µg/mL) for 15 min. The EGFP-tau fluorescence was analyzed in a high-content screening equipment (IN Cell Analyzer 2200, GE Healthcare). For each condition tested, a total of 56 images were acquired from wells, in a 40x magnification. Images were analyzed by the IN Cell Investigator High-Content analysis software v1.3, and data were expressed as the average fluorescence intensity normalized by the control group (Dox^+^).

### Statistics

Comparisons between groups were performed using one or two-way Analysis of Variance (ANOVA) test with the subsequent Dunnett, Tukey or Sidak post-hoc tests when appropriate. For all assessments, the assumed significance level was *p* ≤ 0.05 and data are expressed as the mean ± standard error of the mean (S.E.M.).

## Results

### CBD, ACEA and GW-405,833 Treatments do not Affect Cell Viability

**Fig. 1:**
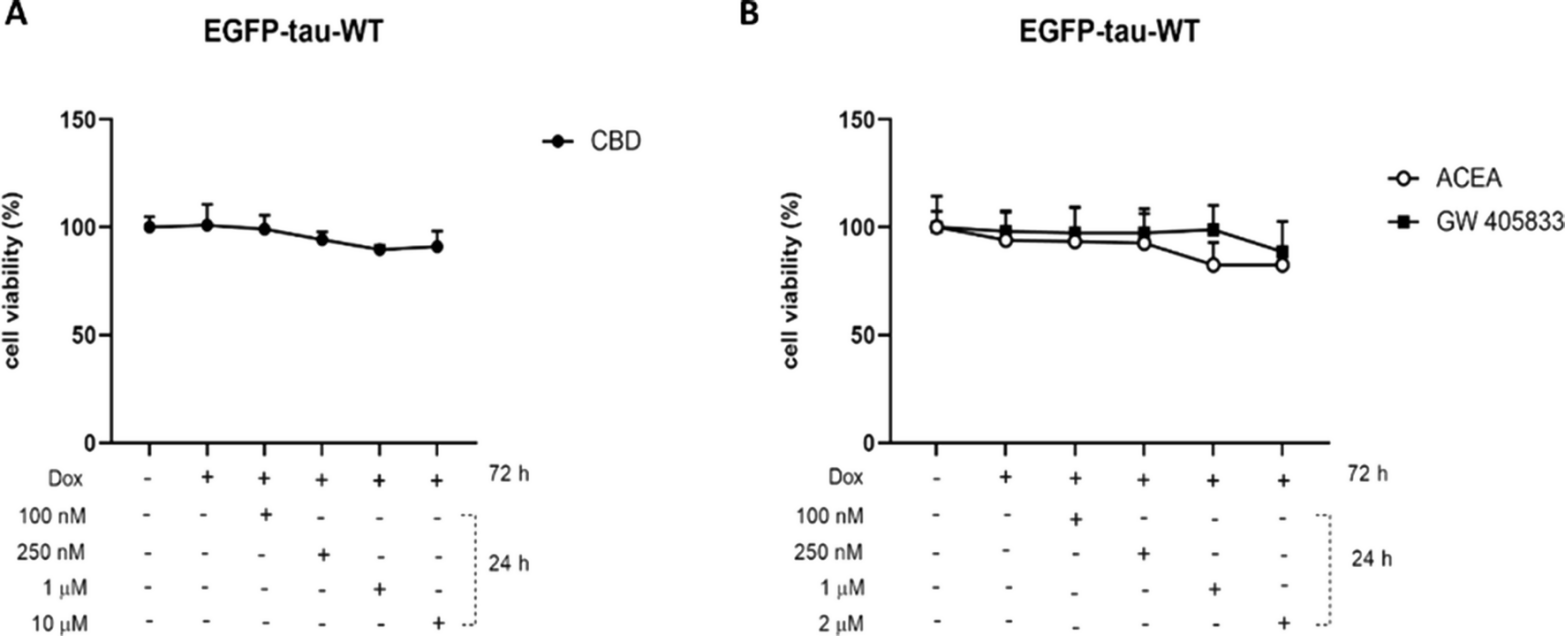
Concentration-response curve of CBD, ACEA and GW-405,833 in the EGFP-Tau WT cell line. Cell viability was obtained from the colorimetric assay after Tet-On system activation with doxycycline (1 μg/mL) for 72 h and treatments with (**A**) CBD (100, 250 nM, 1 and 10 μM), (**B**) ACEA and GW-405,833 (100, 250 nM, 1 and 2 μM) for 24 h. Data are presented as viability of cell modulation in relation to the control without doxycycline. Data are presented as a mean ± SEM of five independent experiments (*n*=5; two-way ANOVA followed by Tukey’s post-test)

To characterize the overexpression of EGFP-Tau WT construct (~ 90 kD), the expression of total tau (Tau-5) was evaluated after 72 h of system activation with doxycycline (1 µg/mL) (Suppl. Figure 1 A-B). Data demonstrated the increase in tau-5 expression in the group treated with doxycycline, when compared to non-treated control group. Immunoblot analysis also showed no variation in endogenous tau when the Tet-On system is activated. After the activation of Tet-On system, the toxicity of cannabidiol and the selective agonists ACEA (CB1) and GW-405,833 (CB2) receptors, were evaluated. As shown in Fig. 1, there were no significant alterations in cell viability in the groups.

### Cannabidiol Induces Tau Clearance

**Fig. 2:**
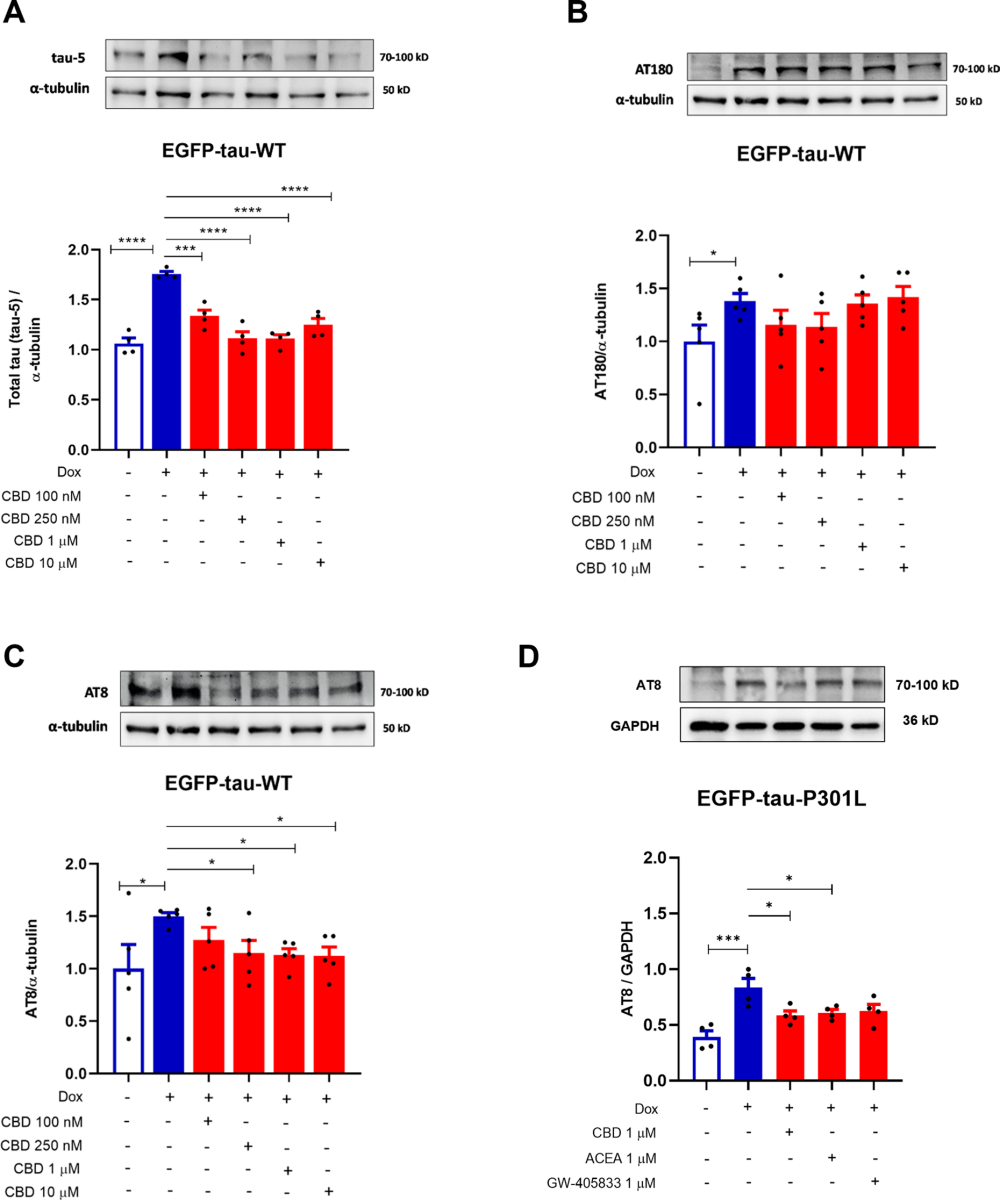
CBD differentially affects the phosphorylation of AT8, AT180 proteins in EGFP-Tau WT and EGFP-Tau-PL301-expressing cell lines. EGFP-Tau WT cells were treated with CBD (100, 250 nM, 1 and 10 µM) for 24 h and cytosolic levels of AT8, AT180 and total tau (Tau-5) were evaluated by western blotting. EGFP-Tau-P301L cells were treated with CBD, ACEA and GW405833 at 1 µM for 24 h and cytosolic levels of AT8 were evaluated by western blotting. Data represent the mean obtained from the quantification of the optical density of the bands obtained by western blotting with the antibodies (**A**) anti-tau-5 (70-100 kD), (**B**) anti-pAT180 (70-100 kD), (**C**-**D**) anti-p-AT8 (70-100 kD) and normalized to the internal control α-tubulin (50 kD) or GAPDH (36 kD), as shown in the histograms (mean ± SEM). *p<0.05 and ****p<0.0001 vs. CTR (Dox^–^) and *p<0.05, ***p<0.001 and ****p<0.0001 vs. CTR (Dox^+^) (*n*=3-5; One-way ANOVA, followed by the Sidak post-test)

Next, after in Tau expressing SH-SY5Y cells (Dox^+^) presented a significant increase of tau-5 and its sites of phosphorylation AT8 (pSer202/pThr205) and AT180 (pThr231) (69% (*p* < 0.001); 50%, *p* = 0.0206 and 38.7%, *p* = 0.0490; respectively), when compared to the control (Dox^−^) (Fig. 2A-C). The results showed that the treatment with CBD significantly decreased the levels the phosphorylation sites at AT8 pSer202/pThr205 (tau AT8) by 35% (*p* = 0.020), 37% (*p* = 0.015) and 38% (*p* = 0.013), at 250 nM, 1 and 10 µM, respectively (Fig. 2A). On the other hand, no significant differences were observed in the AT180 phosphorylation (Fig. 2B). Additionally, CBD was able to decrease total tau (Tau-5) by 42% (*p* < 0.001), 64%, 64% and 50% (*p* < 0.0001) at 100, 250 nM, 1 and 10 µM, respectively, when compared to Dox^+^ (Fig. 2C). These data indicated that activation of Tet-On system induces the accumulation of tau total (Tau-5) and the phosphorylation of AT8 and AT180 proteins. Importantly, all concentrations tested of CBD abrogated the tau-5 and AT8 phosphorylation. Given this, we investigated the effect of CBD and the cannabinoid agonists ACEA and GW405833 on the AT8 phosphorylation site in the EGFP-tau-P301L cell line, which carries a mutated tau protein. Interestingly, CBD and ACEA at 1 µM after 24 h also reduced phosphorylation of AT8 in mutated tau by 30% (*p* = 0.0251) and 27% (*p* = 0.0465), respectively (Fig. 2D).

### CBD Induces Autophagy in Tau WT Expressing SH-SY5Y Cell Line

**Fig. 3:**
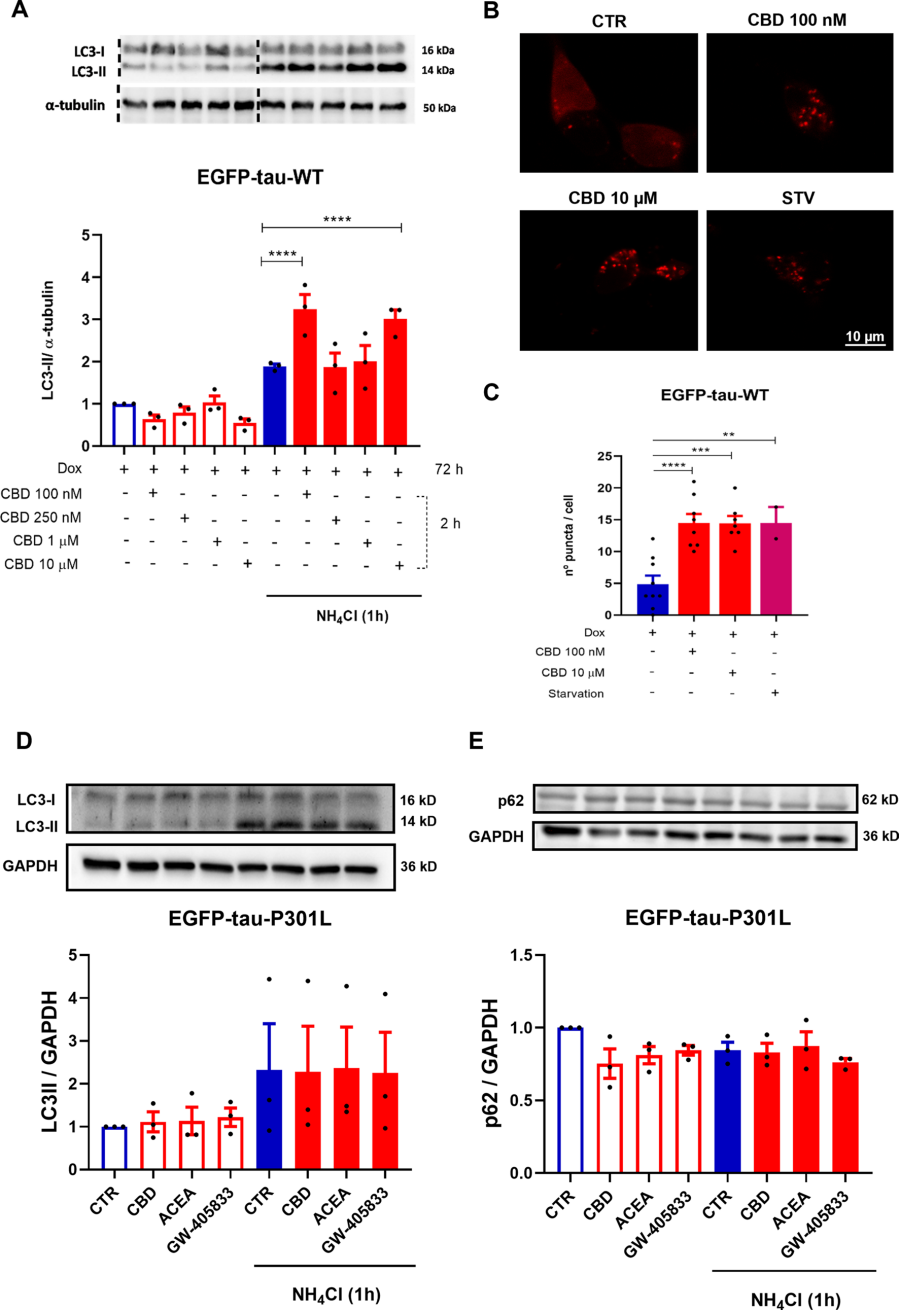
CBD and CB1/2 selective agonists differentially regulates autophagic flux in the EGFP-Tau WT or EGFP-Tau-PL301-expressing cell lines. (**A**) Cytosolic protein extracts were prepared and the autophagic flux was evaluated by western blotting in the EGFP-Tau WT cells treated with CBD (100, 250 nM, 1 and 10 µM) for 2 h in the presence or absence of NH_4_Cl (10 mM), added in the last hour of treatment. Mean LC3-II expression levels were compared between the CBD-treated groups, the control group (no treatment) and the NH_4_Cl group. Data represent the average optical density obtained by western blotting analysis with the anti-LC3-II antibody (15 kD) and normalized with the internal control for the anti-α-tubulin antibody (50 kD) as shown in the histograms (mean ± SEM). ****p<0.0001 vs. NH_4_Cl group (*n*=3; two-way ANOVA, followed by Tukey’s post-test). (**B**) Representative images of the EGFP-Tau WT cell line overexpressing the mCherry-LC3 plasmid and treated with CBD (100 nM and 10 μM) or submitted to nutritional deprivation (starvation– STV) for 2 h. (**C**) Quantification of mCherry-LC3 positive puncta (autophagosomes) after treatments with CBD (100 nM and 10 μM) and STV for 2 h. Images were obtained from at least 6 different fields, in a Carl Zeiss LSM 780 confocal microscope, at 63× magnification. Scale bar 10 μm. mCherry-LC3 Excitation/Emission: 543 / 615 nm Data are expressed as mean ± SEM **p<0.01, ***p<0.001 and ****p<0.0001 vs. CTR (One-way ANOVA, followed by Dunnett’s post-test). Cytosolic protein extracts were prepared and the autophagic flux was evaluated by western blotting in the EGFP-Tau-PL301 cells treated with CBD, ACEA and GW-405,833 (1 µM) for 2 h in the presence or absence of NH_4_Cl (10 mM), added in the last hour of treatment. (**C**) Mean LC3-II and (**D**) p62 expression levels were compared between CBD, ACEA and GW-405,833 treated groups, the control group (no treatment) and the NH_4_Cl group. Data represents the average optical density obtained by western blotting analysis with the anti-LC3-II antibody (15 kDa) or anti-p62 antibody (62 kDa) and normalized to the internal control GAPDH (36 kDa) as shown in the histograms. Data are expressed as mean ± SEM, vs. CTR or NH_4_Cl groups (*n*=3; Two-way ANOVA, followed by Tukey’s post-test)

Furthermore, investigating the role of autophagy in tau degradation mediated by CBD. For this purpose, LC3-II protein was quantified in the EGFP-Tau WT cell line after treatment with CBD (100, 250 nM, 1 and 10 µM) for 2 h. The cells were also treated in presence and absence of ammonium chloride (NH_4_Cl, 2 mM), an inhibitor of the final step of autophagic flux, added in the last hour of treatment. As demonstrated in the representative immunoblots, the groups treated with CBD 100 nM and 10 µM had a further increase in LC3-II accumulation, 135.9% and 112.4% (*p* < 0.0001), when compared to NH_4_Cl (Fig. 3A). To evaluate the potential of CBD in inducing autophagy, the EGFP-Tau WT cell line overexpressing mCherry-LC3 was treated with CBD at 100 nM and 10 µM for 2 h, as previously demonstrated to activate autophagy. As a positive control, the cells were subjected to nutritional deprivation (starvation - STV) by Earle’s Balanced Salt Solution (EBSS), also for 2 h. The number of autophagosomes was performed in single-cells analysis under a confocal microscope (Fig. 3B). The data showed that there was an increase of mCherry puncta (LC3-II) of 96.1% (*p* < 0.0001), 95.4% (*p* = 0.0001) and 96.1% (*p* = 0.0097), in the CBD (100 nM), CBD (10 µM) and STV treated groups, respectively, when compared to the control (Dox^+^) (Fig. 3C). At the same concentrations, that CBD increased the levels of LC3-II protein in the immunoblot, it also induced the formation of autophagosome, which were observed in cells transfected with mCherry-LC3. In addition, to investigate the effect of CBD on autophagy in mutated tau, SH-SY5Y cells expressing EGFP-Tau-P301L were used. In contrast to prior findings, CBD and the selective cannabinoid agonists ACEA and GW-405,833 at 1 µM for 2 h did not modulate the levels of LC3-II (*p* = 0.9844) or p62 (*p* = 0.9749) (Fig. 3D-E, respectively).

### CBD-Induced Autophagy Reduces Tau Accumulation Via Autophagy

**Fig. 4:**
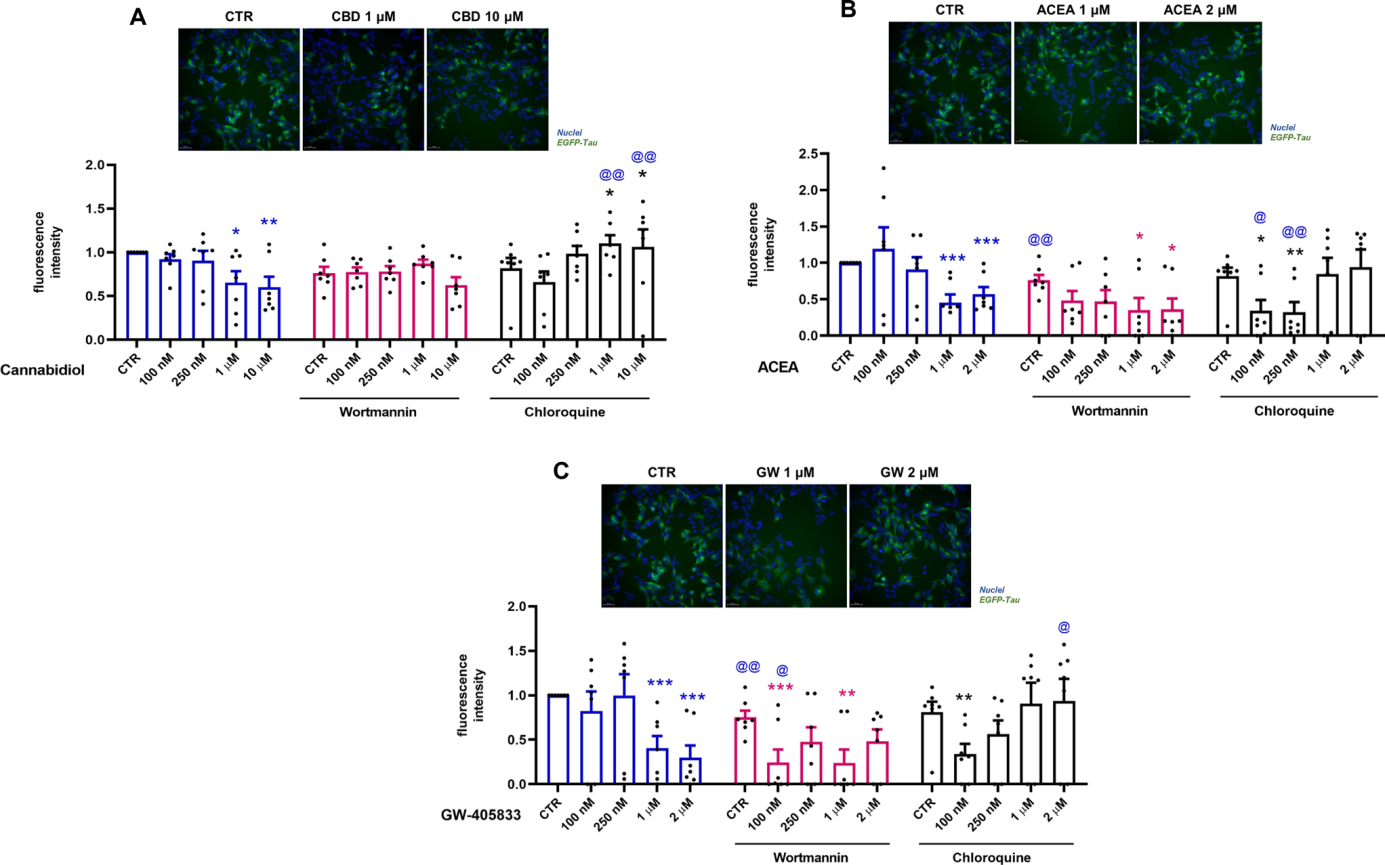
EGFP-Tau fluorescence intensity levels modulated by CBD, ACEA and GW-405,833 in the presence and absence of autophagic inhibitors. Cells were activated with doxycycline (1 µg/mL) for a period of 72 h, pre-treated with wortmannin (250 nM) and chloroquine (25 µM) for 30 min and then treated with CBD (100, 250 nM, 1 and 10 µM), ACEA (100, 250 nM, 1 and 2 µM) and GW-405,833 (100, 250 nM, 1 and 2 µM) for 24 h. Representative images for CBD (1 and 10 µM), ACEA (1 and 2 µM) and GW-405,833 (1 and 2 µM) were shown. (**A**) Data represent the means of tau fluorescence intensity after treatment with CBD, obtained by the analysis and normalized with the control group (mean ± SEM) *p<0.05 and **p<0.01 vs. CTR group; @@*p*<0.01 vs. respective group without inhibitors (*n*=7; two-way ANOVA, followed by the Sidak post-test). (**B**) Data represent the means of tau fluorescence intensity after treatment with ACEA obtained by the analysis and normalized with the control group (mean ± SEM). *p<0.05 and **p<0.01 vs. CTR group; @*p*<0.05 and @@*p*<0.01 vs. respective group without inhibitors (*n*=7; two-way ANOVA, followed by the Sidak post-test). (**C**) Data represent the means of tau fluorescence intensity after treatment with GW-405,833 obtained by the analysis and normalized with the control group (mean ± SEM). *p<0.05, **p<0.01 and ***p<0.001 vs. CTR group; @*p*<0.05 and @@*p*<0.01 vs. respective group without inhibitors (*n*=7; two-way ANOVA, followed by the Sidak post-test)

As previously demonstrated, CBD induces autophagy and a tau reduction after 2 and 24 h of treatment. Using selective CBR agonists, EGFP-Tau WT-overexpressing cells were treated at different concentrations CBD, ACEA and GW405833 in association with the autophagic inhibitors: wortmannin (250 nM) and chloroquine (25 µM). Wortmannin is a specific inhibitor of phosphoinositidine 3-kinases (PI3Ks), used as an inhibitor of autophagy since it inhibits the initiation of the autophagosome (HUNG et al., 2009). Chloroquine is an autophagic blocker since it interferes in the fusion of autophagosome and lysosome. Thus, after the activation of Tet-On system, cells were pretreated with the autophagic inhibitors for 30 min, followed by CBD treatment and the selective agonists of CB1 and CB2, ACEA and GW405833 (1 and 2 µM), respectively for 24 h. The quantification of GFP fluorescence demonstrated that CBD (1 and 10 µM) decreased the fluorescence intensity of EGFP-Tau WT by 34.8% (*p* = 0.014) and 39.9% (*p* = 0.004), respectively, when compared to the control (Dox^+^) (Fig. 4A). The association of CBD with wortmannin did not affect the fluorescence intensity when compared to the control group. The groups co-treated with chloroquine increased 45% and 46% (*p* = 0.005) in fluorescence intensity in the groups treated with 1 µM and 10 µM CBD, respectively. These data demonstrated that chloroquine blocked the autophagic flux, causing the accumulation of tau and, consequently, increased the intensity of GFP fluorescence.

Cells treated with ACEA showed a significant decrease of 54.6% and 42.9% (*p* = 0.001) in the fluorescence intensity of EGFP-Tau WT at 1 and 2 µM, respectively (Fig. 4B). The association of ACEA and wortmannin caused a decrease of 23.9% (*p* = 0.004) of EGFP-Tau WT fluorescence when compared to control. Additionally, wortmannin at 1 and 2 µM led to a decrease of 41% (*p* = 0.027) and 40% (*p* = 0.025) in fluorescence intensity in relation to the control, respectively.

The co-treatment with chloroquine decreased in 47.7% (*p* = 0.017) and 49.5% (*p* = 0.005) compared to the control and 85.2% (*p* = 0.017) and 58.6% (*p* = 0.010) at 100 and 250 nM, respectively.

Cells treated with GW-405,833 showed a significant decrease of 59.3% and 69.9% (*p* = 0.001) in the EGFP-Tau WT fluorescence intensity at 1 and 2 µM, respectively (Fig. 4C). Co-treatment of GW-405,803 with wortmannin led to a further decreased of 51% (*p* = 0.001) e 51.1% (*p* = 0.0.007) in fluorescence intensity in relation to the wortmannin control, at 100 nM and 1 µM, respectively. Also, GW-405,803 was able to decrease 24.8% (*p* = 0.002) and 75.8% (*p* = 0.027) in relation to their respective groups without inhibitors, control group and group treated with 100 nM, respectively. The co-treatment with chloroquine showed a reduction of 47% (*p* = 0.010) in relation to chloroquine alone and an increase of 93.03% (*p* = 0.034) in the group treated with 2 µM in relation to its non-treated group (Fig. 4C). In summary, these data showed that the cannabinoids CBD, ACEA and GW-405,833 decreased the intensity of tau fluorescence. However, when using the autophagic inhibitors, wortimanin and chloroquine, only the CBD (1 and 10 µM) and GW-405,833 (2 µM) + chloroquine groups, reversed the decrease in the intensity of EGFP-Tau WT, demonstrating the possible participation of autophagy in the degradation of tau in these groups.

## Discussion

Several studies have reported a possible crosstalk between the tau hyperphosphorylation and autophagy dysfunctions (Nassif and Hetz 2012; Silva et al. 2019; Di Meco et al. 2020). Tau hyperphosphorylations were found in Atg7 knockout mouse brains, which was reverted after the restoration of autophagy (Inoue et al. 2012). Besides the assembly and the stabilization of microtubules, tau protein is crucial for the maintenance of retrograde traffic and maturation of autophagosomes, as well as their fusion with lysosomes (Dickey et al. 2007). In fact, tau hyperphosphorylation can lead to instability of the microtubule cytoskeleton, which can inhibit autophagosome traffic and favor the accumulation of immature autophagosomes in axons (Nassif and Hetz 2012; Piras et al. 2016).

Increasing evidence indicate that the activation of CB1 and CB2 receptors, by natural or synthetic agonists, mediates neuroprotective effects in in vitro and in vivo models of AD, reducing the deleterious effects of βA peptide or tau hyperphosphorylation (Esposito et al. 2007; Martin-Moreno et al. 2011; Janefjord et al. 2014; Ahmed et al. 2015). Furthermore, Esposito et al. (2006) showed that CBD (from 100 nM to 10 µM) inhibited tau hyperphosphorylation in PC12 cell line stimulated by βA peptide (Esposito et al. 2006).

Here the high levels of AT8 levels in EGFP-Tau WT cells and in the mutated tau cell line EGFP-Tau-P301L were attenuated by the treatment with CBD (250 nM, 1 or 10 µM) for 24 h. These results showed that the Tet-On system activation induced tau AT8 on pSer202/pThr205 sites, and CBD treatment decreased these phosphorylations, indicating a potential neuroprotective effect. Interestingly, CBD (100, 250 nM, 1 and 10 µM, 24 h) was able to decrease total tau, strongly suggesting a total tau clearance. This evidence was reported in other studies, in mice overexpressing human tau (PK^−/−^TauVLW), a frontotemporal complex model, Sativex^®^ induced a marked reduction in neurofibrillary tangles (Casarejos et al. 2013).

However, it was not possible to observe a decrease in AT180 phosphorylation after treatment with CBD (100, 250 nM, 1 and 10 µM, 24 h). The pThr231 (Tau AT180) was observed only in some neurofibrillary tangles, most of which were found in amyloid plaques in the cortex of patients with AD and FTD (Spillantini et al. 1996). However, a study using a transgenic model in zebrafish, expressing human tau P301L, has demonstrated that AT180 is present in tau hyperphosphorylation, but there was not related to neurofibrillary tangles formation (Cosacak et al. 2017).

Several studies have suggested that cannabinoid compounds (Δ9-THC and CBD) potentiated autophagy reducing the deposition of tau proteins. Piras et al. (2016) showed that hyperphosphorylated tau was co-localized on surfaces labeled for LC3-II and p62 proteins in the brain of patients with progressive supranuclear palsy and corticobasal degeneration (Piras et al. 2016). Our results demonstrated autophagy activation in cells overexpressing EGFP-Tau WT cells after treatment with CBD (100 nM and 10 µM, 2 h). This result was confirmed in EGFP-Tau WT cells transfected with the mCherry-LC3, indicating an increase in the number of puncta, indicative of autophagosome formation.

Next, to evaluate the participation of autophagy on tau expression, the EGFP-Tau WT cells were treated with the cannabinoid compounds CBD, ACEA and GW-405,833 with the pre-treatment with classic autophagic inhibitors wortmannin or chloroquine. The use of ACEA (CB1 agonist) and GW-405,833 (CB2 agonist) stems from their ability to provide targeted insights into the specific pathways mediated by cannabinoid receptor activation. Unlike CBD, which interacts with multiple biological targets, these selective agonists enable a focused evaluation of the roles of CB1 and CB2 in autophagy and tau clearance, complementing the broader effects observed with CBD. The results showed that the cannabinoid compounds: CBD (1 and 10 µM), ACEA and GW-405,833, at 1 and 2 µM, were able to significantly reduce the intensity of fluorescence in the EGFP-Tau WT cells. Libro et al. (2017) also observed that CBD led to the downregulation of genes linked to AD, including genes that encode kinases responsible for phosphorylation of tau (Libro et al. 2017). Furthermore, other studies observed that ACEA (CB1 agonist) and WIN55,212-2 (CB1/CB2 agonist) decreased tau phosphorylation in mouse models of AD (Esposito et al. 2006; Aso et al. 2012). We also investigated the effects of CBD on autophagy modulation in mutated tau-P301L. CBD, ACEA, and GW-405,833 were found to block autophagy flux in this cell line, as reported by Costa et al. (2022). Then, in order to evaluate the role of autophagy in tau clearance mediated by cannabinoid compounds, EGFP-Tau WT cells were treated with wortmannin (500 nM, 30 min) prior of treatment with different concentrations of CBD, ACEA and GW-405,833. Wortmannin, a non-specific inhibitor of PI3K-related enzymes (Feldman and Shokat 2010), consequently inhibiting the autophagosome formation (Hung et al. 2009). Interestingly, the wortmannin-only group had a significant decrease in EGFP-Tau WT fluorescence intensity. Thus, the cannabinoid compounds ACEA and GW-405,833 decreased the intensity of tau fluorescence, but in an mTOR-independent manner, since wortmannin inhibits PI3K enzymes related to this pathway. These data corroborate previous data published by our group, that shows that CBD activates autophagy in a mTOR-independent manner (Vrechi et al. 2021). However, how wortmannin increased the intensity of tau (hyperphosphorylation) in EGFP-Tau WT cells have not been elucidated. In addition, the EGFP-Tau WT cells were also treated with chloroquine. Of note, groups treated with chloroquine and CBD (1 and 10 µM) increased GFP intensity. These results suggested CBD was able to decrease EGFP-Tau WT via autophagy, since chloroquine potentially inhibit the lysosomal degradation, the last step of autophagic process. One study revealed that chloroquine (50 µM) induced the accumulation of autophagic vacuoles and blocked autophagic outflow in retinoic acid-differentiated SH-SY5Y cells (Pivtoraiko et al. 2010). Taken together, the collecting data provides evidence that CBD was able to reduce total tau via AT8 pSer202/pTrh205 dephosphorylation and autophagy activation. Moreover, the cannabinoid compounds CBD, ACEA and GW-504,833 were able to decrease the fluorescence intensity of the EGFP-Tau WT, demonstrating the possible protein degradation in this model. Furthermore, in the presence of chloroquine, an autophagic inhibitor, the treatment with CBD (1 and 10 µM) and GW-405,833 (2 µM) reversed tau clearance, indicating the participation of autophagy in tau degradation in these groups.

## Conclusion

CBD induces autophagy promoting tau clearance in an in vitro model of tauopathy. Moreover, CBD, ACEA and GW-405,833 decreased tau expression, which was reversed by chloroquine indicating that autophagy participates in tau clearance. Our results support the relevance of cannabinoid compounds in the autophagic process involved in the degradation of accumulated tau, which has been associated with several neuropathies. Therefore, autophagy is a potential therapeutic target of cannabinoids in neurodegenerative diseases.

## Electronic Supplementary Material

Below is the link to the electronic supplementary material.

Supplementary Material 1

## Data Availability

No datasets were generated or analysed during the current study.
