# Supplementary figures and images for: Cannabidiol-Induced Autophagy Ameliorates Tau Protein Clearance
Source: Neurotox Res. 2025 Feb 4;43(1):8. doi: 10.1007/s12640-025-00729-3 (PMC11790692; doi:10.1007/s12640-025-00729-3)

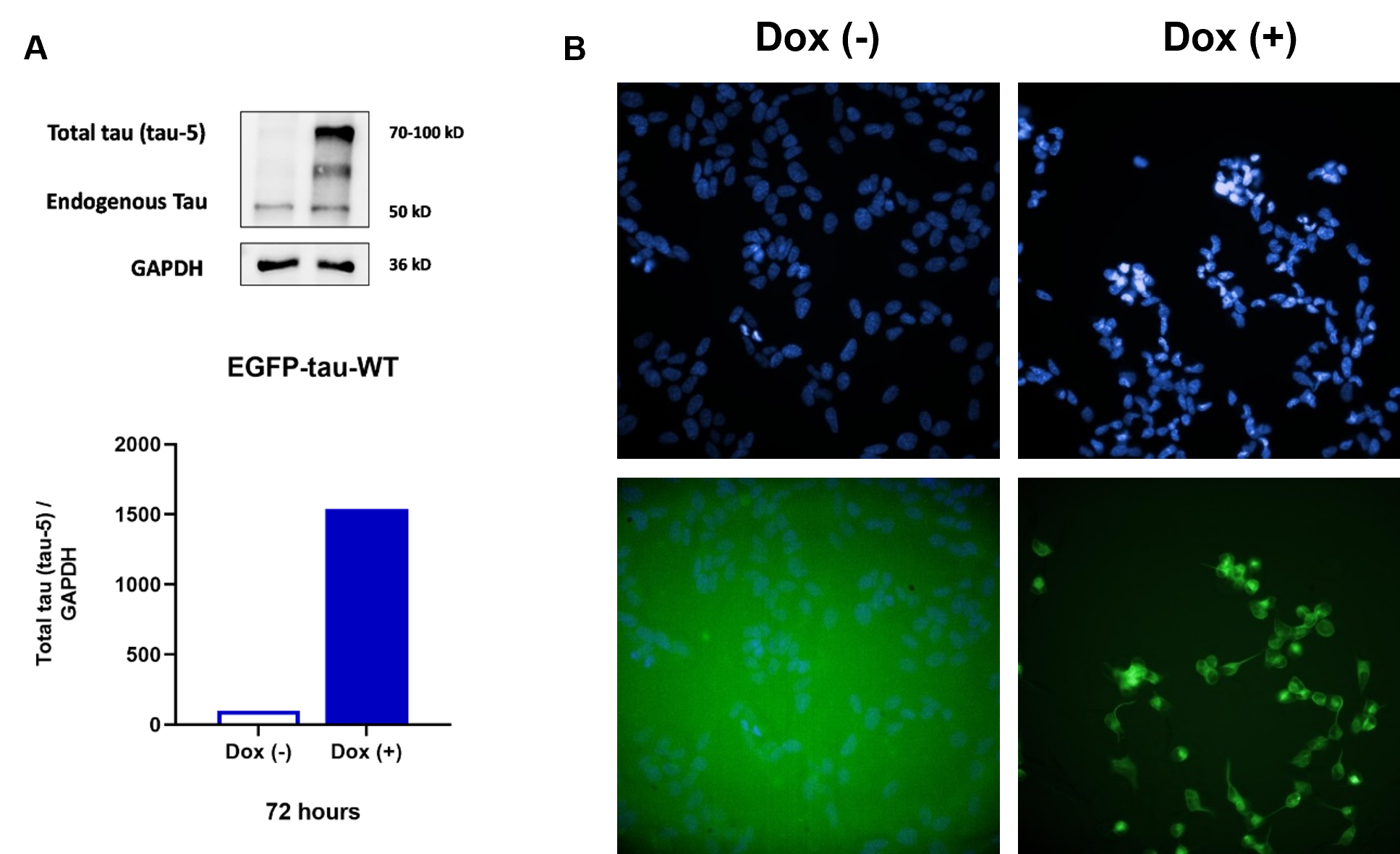

Supplement: Supplementary file 1 — Supplementary Material 1 [file 12640_2025_729_MOESM1_ESM.tif]
